# Supplementary material for: Enzymatic Spermine Metabolites Induce Apoptosis Associated with Increase of p53, caspase-3 and miR-34a in Both Neuroblastoma Cells, SJNKP and the N-Myc-Amplified Form IMR5
Source: Cells. 2021 Jul 31;10(8):1950. doi: 10.3390/cells10081950 (PMC8393918; doi:10.3390/cells10081950)
Supplement: Supplementary file 1 [file cells-10-01950-s001.zip › cells-1310952 supplementary.pdf]

Figure S1 Western blotting of SJNKP

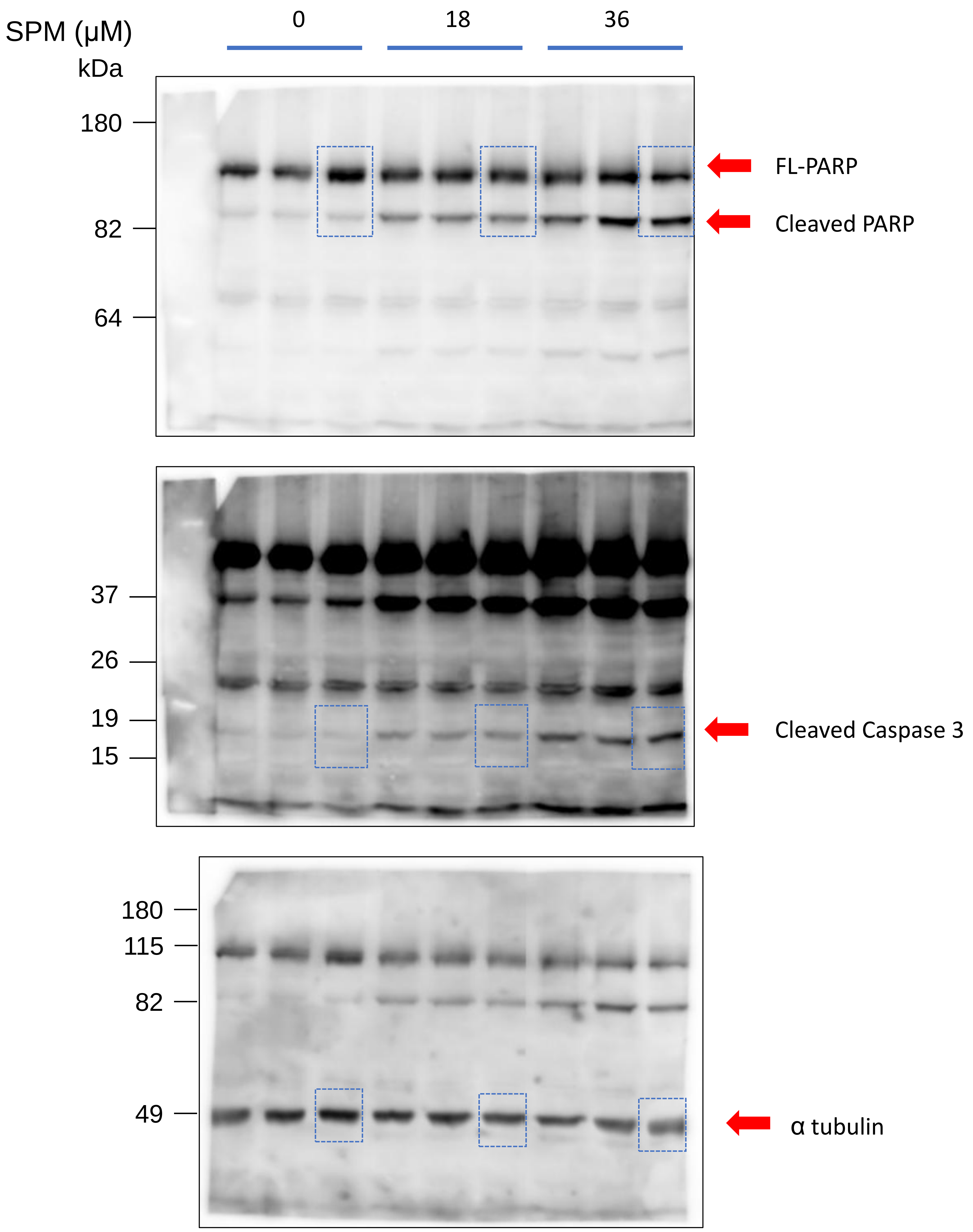

Figure 7A raw data

Figure S2 Western blotting of IMR5

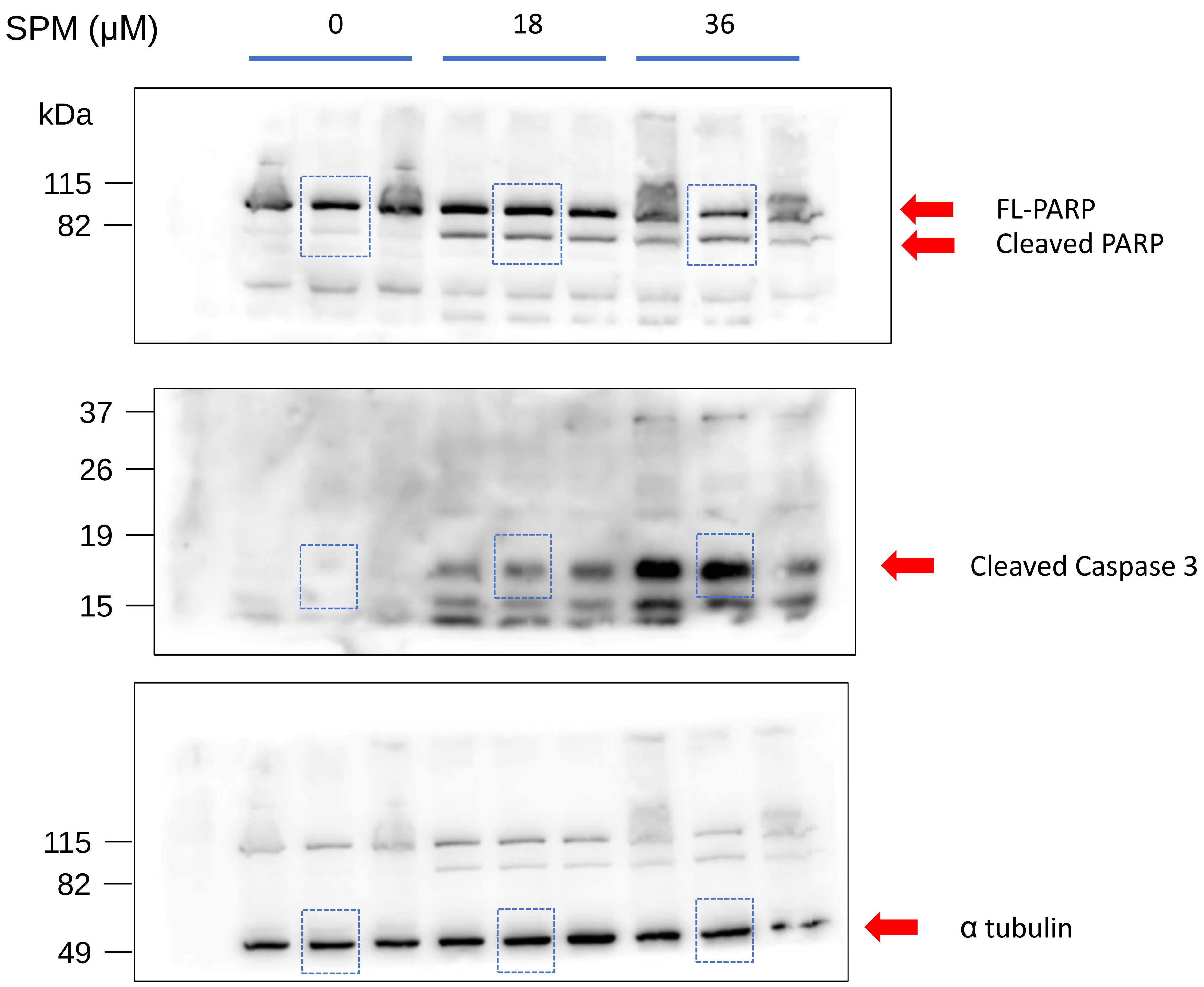

Figure 7C raw data
